# Associations of TyG-Derived Indices with Cardiometabolic Multimorbidity Risk in Community-Dwelling Older Adults: A Longitudinal Analysis Based on the GOLD-Health Cohort

**DOI:** 10.3390/nu18060985

**Published:** 2026-03-19

**Authors:** Chuming Liao, Hui Liu, Suqi Xu, Zhen Ling, Yue Zhuo, Guihua Huang, Weiquan Lin, Zhoubin Zhang

**Affiliations:** 1 School of Public Health, Southern Medical University, Guangzhou 510515, China; missaka159@163.com (C.L.); xusuqi1011@163.com (S.X.); 2 Department of Basic Public Health, Guangzhou Center for Disease Control and Prevention (Guangzhou Health Supervision Institute), Guangzhou 510440, China; gzcdc_liuhui@163.com (H.L.); gzcdc_lingz@163.com (Z.L.); zhuoyuegirl@163.com (Y.Z.); hgh_huangguihua@163.com (G.H.); 3 Institute of Public Health, Guangzhou Medical University, Guangzhou Center for Disease Control and Prevention (Guangzhou Health Supervision Institute), Guangzhou 510440, China

**Keywords:** triglyceride-glucose index, insulin resistance, cardiometabolic multimorbidity, older adults, cohort study, predictive markers

## Abstract

Background/Objectives: Cardiometabolic multimorbidity (CMM) significantly reduces healthy life expectancy in older adults. The specific role of adiposity indices derived from the triglyceride-glucose (TyG) index, body mass index (BMI), and waist-to-height ratio (WHtR) in predicting incident CMM has not been fully elucidated in longitudinal settings. We investigated these associations and the mediating role of the atherogenic index of plasma (AIP). Methods: We analyzed 304,586 community-dwelling adults aged ≥65 years from the prospective Guangzhou Older Longitudinal Dynamic Health (GOLD-Health) cohort (2018–2019), who were free of CMM at baseline. Multivariable Cox proportional hazards models evaluated the risk of incident CMM (coexistence of ≥2 cardiometabolic diseases) across quartiles of six TyG-derived indices. Mediation analysis quantified the contribution of atherogenic dyslipidemia via AIP. Results: Following a median observation time of 4.3 years, the study recorded 7816 participants who developed CMM. All six indices showed significant positive associations with CMM risk. TyG-WHtR demonstrated the strongest association (Hazard Ratio [HR] comparing highest vs. lowest quartile = 2.150; 95% Confidence Interval [CI] 1.998–2.314), closely followed by TyG-BMI (HR = 2.146). AIP significantly mediated the associations, explaining 7.5–33.0% of the effect, with the highest proportion observed for TyG using the Chinese visceral adiposity index (CVAI). Conclusions: TyG-derived adiposity indices, particularly TyG-WHtR and TyG-BMI, are robust independent risk markers for incident CMM in older adults. The substantial mediating role of AIP suggests that targeting atherogenic dyslipidemia may be a key strategy to interrupt the progression from insulin resistance to multimorbidity. These accessible metrics hold promise for large-scale risk stratification and early intervention in primary care settings.

## 1. Introduction

With the acceleration of population ageing, multimorbidity is increasingly becoming the norm [1]. Cardiometabolic multimorbidity (CMM) represents a distinct and severe pattern within the broader spectrum of multimorbidity. Currently, it is recognized as a major global health challenge. This condition is defined by the concurrent presence of at least two cardiometabolic diseases (CMDs), specifically including hypertension, diabetes mellitus (DM), coronary heart disease (CHD), and stroke [2,3,4]. Compared with individuals suffering solely from single CMD, patients with CMM face a multiplicative rather than additive effect on risk of mortality, resulting in a significantly reduced life expectancy. For instance, the onset of CMM at age 60 is associated with a 12 to 15-year reduction in life expectancy [5]. Due to the irreversible nature and detrimental effects of confirmed CMM, identifying modifiable risk factors and sensitive biomarkers in healthy individuals or those with isolated CMD is crucial for early intervention and prevention within primary healthcare settings.

Bacci and Bao showed that insulin resistance (IR) is a shared root for diabetes and heart disease [6,7]. They used inflammation and genetic markers to support this ‘common soil’ idea. Based on this framework, we focus on IR as a common pathological mechanism underlying various metabolic disorders [8,9,10,11,12]. The TyG index serves as a practical surrogate marker for IR because it is derived from fasting glucose and lipid profiles. However, the TyG index alone does not account for body fat distribution. Recent research indicates that composite indicators like TyG-BMI and TyG-WHtR may reflect the combined effects of IR and visceral fat more effectively [12,13,14,15,16]. Many studies already show that these indices are linked to single cardiovascular outcomes [13,17]. However, we still need more data on how TyG-derived fat indices affect the accumulation of multiple diseases in older adults.

Furthermore, the biological pathways linking these composite adiposity-IR indices to the development of CMM are not fully understood. Atherogenic dyslipidemia, often quantified by the atherogenic index of plasma (AIP), is a key driver of vascular damage, yet its potential mediating role in this relationship has not been explored. Based on the GOLD-Health cohort, our primary objective was to determine the longitudinal impact of six TyG-derived indices on the risk of incident CMM. Additionally, we examined the mediating role of AIP to facilitate better risk stratification and mechanistic understanding in older adults.

## 2. Materials and Methods

### 2.1. Study Design and Population

Data were derived from the Guangzhou Older Longitudinal Dynamic Health (GOLD-Health) cohort [18], a large-scale prospective study designed to monitor health dynamics among community-dwelling older adults within the national basic public health service framework. Adhering to standardized protocols, trained clinicians conducted baseline data collection via face-to-face interviews, physical examinations, anthropometric measurements, and laboratory tests. This study was reviewed and approved by the Ethics Committee of the Medical Ethics Research Center of Guangzhou Center for Disease Control and Prevention (GZCDC-ECHR-2023P0081) and registered by the China Clinical Trial Registry (ChiCTR2400089945).

**Figure 1:**
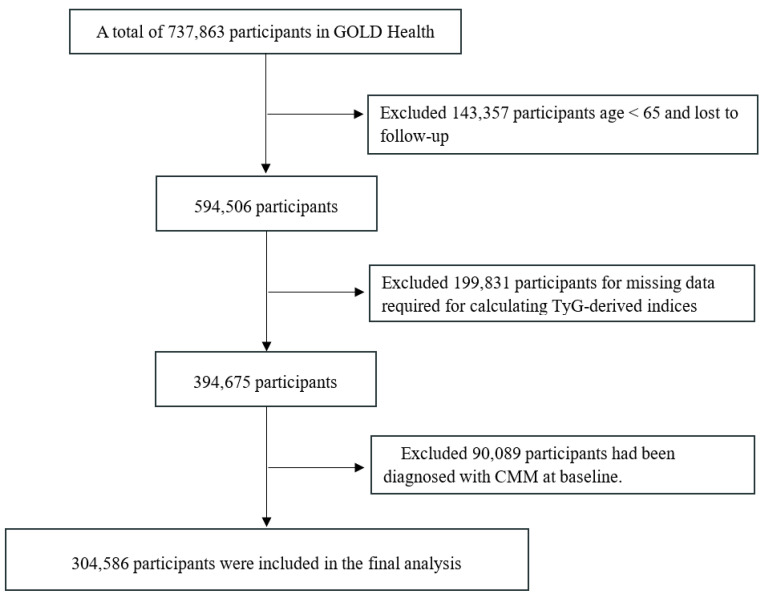
Flowchart of selecting study population in the GOLD-Health cohort.

We recruited participants aged 65 years or older between 2018 and 2019. From an initial pool of 737,863 individuals with baseline blood test records, we excluded those who were under 65 or already had CMM at baseline. Additionally, participants were removed if they had missing data on key variables (such as gender, lipid profiles, and body measurements) or were lost to follow-up. Consequently, a total of 304,586 subjects were included in the final analysis. Please refer to Figure 1 for the detailed screening flow.

#### 2.1.1. Exposures and Outcome

The following formulas were used for TyG and its derived indices [19,20,21]:

Triglyceride glucose (TyG) = ln [TG (mg/dL) × FBG (mg/dL)/2];
Body mass index (BMI) = Weight(kg)/Height^2^(m^2^);
A body shape index (ABSI) = WC (cm)/[BMI (kg/m^2^)^2/3^ × height ^1/2^ (cm)];
Weight-adjusted waist index (WWI) = WC (cm)/Weight^1/2^(kg);
Chinese visceral adiposity index (CVAI):
For male: CVAI = −267.93 + 0.68 × age (years) + 0.03 × BMI (kg/m^2^) + 4.00 × WC (cm) + 22.00 × lg [TG (mmol/L)] − 16.32 × HDL-C(mmol/L);
For female: CVAI = −187.32 + 1.71 × age (years) + 4.23 × BMI (kg/m^2^) + 1.12 × WC (cm) + 39.76 × lg [TG (mmol/L)] − 11.66 × HDL-C (mmol/L);
Waist–height ratio (WHtR) = WC (cm)/height (cm);
Body roundness index (BRI) = 364.2 − 365.5 × [1 − (0.01 × WC (cm)/(2 × *π*)/0.5/height(m))^2^] ^1/2^.

The primary endpoint event of this study was the occurrence of CMM events, characterized by the coexistence of two or more types of CMD, including hypertension, DM, heart disease and stroke [4,22,23]. For hypertension, participants were identified based on blood pressure measurements (SBP ≥ 140 mmHg or DBP ≥ 90 mmHg), a self-reported physician diagnosis, or the current use of antihypertensive medication. Similarly, DM was determined by an FBG ≥ 126 mg/dL, HbA1c ≥ 6.5%, a prior medical diagnosis, or antidiabetic drug usage. We classified participants with FBG levels of 100–125 mg/dL or HbA1c of 5.7–6.4% as having pre-diabetes, while those below these thresholds were considered to have normal glucose regulation [24,25]. Regarding CVD outcomes like CHD or stroke, identification relied on clinical diagnoses reported by physicians during follow-up examinations. We also verified self-reported medical history by asking participants about specific diagnoses and dates. The follow-up duration was calculated from the date of enrollment. It ended on the date of the first confirmed CMM event. For participants who remained free of CMM, the observation period ended at the date of the final survey [18].

#### 2.1.2. Covariates

We considered a wide range of covariates in this analysis. Sociodemographic and lifestyle variables included age, gender, education status, marital status, smoking status, drinking status, and physical activity (PA). Body measurements consisted of height, weight, waist circumference (WC), body mass index (BMI), systolic blood pressure (SBP), and diastolic blood pressure (DBP). We also collected information on disease history regarding conditions such as cancer, chronic obstructive pulmonary disease (COPD), and hyperlipoidemia. Additionally, we recorded medications taken for hypertension, hyperlipoidemia, and DM. Finally, the laboratory data covered fasting blood glucose (FBG), total cholesterol (TC), triglycerides (TG), high-density lipoprotein cholesterol (HDL-C), low-density lipoprotein cholesterol (LDL-C), serum creatinine (SCr), alanine aminotransferase (ALT), and aspartate aminotransferase (AST).

#### 2.1.3. Statistical Analysis

Participants were stratified into two groups: the CMM group and the non-CMM group. For continuous variables with a normal distribution, we reported values as means with standard deviations (SD). These were compared using one-way ANOVA. Conversely, non-normally distributed data were expressed as medians with interquartile ranges (IQR). We applied the Kruskal–Wallis test to analyze differences for these skewed variables. For categorical data, comparisons were performed using the chi-square (*χ*^2^) test. To evaluate the impact of TyG-derived indices, we categorized these metrics into quartiles. The lowest quartile (Q1) served as the reference group. We then calculated hazard ratios (HRs) and 95% confidence intervals (CIs) using Cox proportional hazards regression models. Three models were constructed. Model 1 was unadjusted. Model 2 included adjustments for age and gender. Finally, Model 3 was fully adjusted for all aforementioned covariates based on Model 2.

Kaplan–Meier curves and log-rank tests were used to assess the cumulative incidence of CMM between quartiles of different indices. Additionally, fully adjusted restricted cubic spline (RCS) analyses were performed to explore the dose–response relationships between the indices and the risk of incident CMM. The receiver operating characteristic (ROC) curves were established to assess the predictive value of these indices for CMM, and the area under the ROC curve (AUC) values were used to evaluate the incremental effect of those indices.

In order to verify the robustness of our findings, we conducted four sensitivity analyses. In sensitivity analysis 1, we excluded participants on antihyperglycemic, antilipidemic, or antihypertensive treatment at baseline to minimize confounding by indicated medication use. In sensitivity analysis 2, we excluded participants with less than one year of follow-up, to reduce the impact of overly short follow-up periods. In sensitivity analysis 3, we restricted the population to participants without any cardiometabolic or chronic diseases at baseline. This approach aimed to minimize the influence of pre-existing health conditions. It allowed for a clearer assessment of the dose–response relationship between TyG-derived indices and incident CMM in a healthy baseline population. In sensitivity analysis 4, we excluded participants aged 85 years or older at baseline to mitigate the influence of age-related heterogeneity. To assess potential heterogeneity across populations, we performed subgroup analyses and interaction tests. Stratification was based on age, distinguishing between those younger than 75 years and those aged 75 years or older. We also included gender and educational attainment. Furthermore, we examined lifestyle factors, specifically categorizing participants by smoking status, alcohol consumption, and physical activity levels.

To investigate the underlying mechanisms, we performed mediation analysis following VanderWeele’s framework for survival data [26]. This approach allowed us to calculate the total, direct, and indirect effects. We also determined the proportion of mediation. To ensure robustness, we generated 95% confidence intervals based on 1000 bootstrap resamples. Previous evidence suggests that AIP links insulin resistance to cardiovascular outcomes [27,28,29,30,31]. Therefore, we identified AIP as a potential mediator in the pathway from visceral obesity to CMM onset. Finally, all statistical computations were carried out using R software (version 4.5.0). Statistical significance was defined as a *p*-value lower than 0.05.

## 3. Results

### 3.1. Population Characteristics

Table 1 summarizes the baseline characteristics of the 304,586 participants, stratified by CMM status. The average age of the cohort was 71.98 years, and males accounted for 39% (*n* = 120,154). Participants who developed CMM differed significantly from those who remained disease-free. Specifically, the CMM group had a higher proportion of females, never-smokers, and alcohol consumers. They also exhibited higher levels of physical activity but lower educational attainment. Regarding comorbidities, the prevalence of pre-diabetes, hyperlipidemia, and hypertension was elevated in the CMM group. Biochemically, these individuals presented with higher SCr, LDL-C, and FBG levels, whereas HDL-C levels were lower. Notably, baseline levels of all six TyG-derived indices were markedly higher in the CMM group compared to the non-CMM group.

### 3.2. Associations Between TyG-Derived Indices and the Risk of CMM

**Figure 2:**
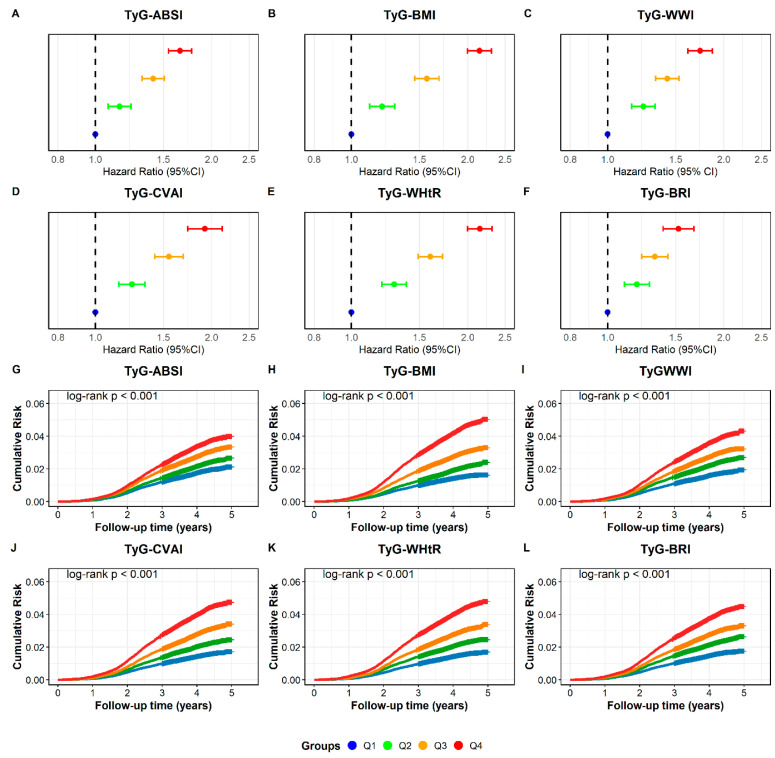
Cardiometabolic multimorbidity incidence across quartiles of six TyG-derived indices with forest plots of Cox regression and Kaplan–Meier survival curves. Figure (**A**–**F**) show forest plots for TyG-ABSI, TyG-BMI, TyG-WWI, TyG-CVAI, TyG-WHtR, and TyG-BRI. In these plots, solid dots represent the HR point estimates. Horizontal error bars indicate the 95% CI. The vertical dashed line represents the reference value of 1.0. Figure (**G**–**L**) show the Kaplan–Meier curves depicting the cumulative incidence of CMM across quartiles of the TyG-derived indices. Note: Forest plots show hazard ratios from three sequential Cox models: model 1 was unadjusted; model 2 was adjusted for age and gender; model 3 added education status, marital status, smoking status, drinking status, PA, BMI, ALT, AST, TC, LDL-C, HDL-C, SBP and DBP, SCr, COPD, cancer, hyperlipidemia, and use of antihypertensive or glucose-lowering medications, while excluding the components of the corresponding TyG-derived index. Abbreviations: PA, physical activity; BMI, body mass index; ALT, alanine aminotransferase; AST, aspartate aminotransferase; TC, total cholesterol; LDL-C, low-density lipoprotein cholesterol; HDL-C, high-density lipoprotein cholesterol; SBP, systolic blood pressure; DBP, diastolic blood pressure; SCr, serum creatinine; COPD, chronic obstructive pulmonary disease; HR, hazard ratio; CI, confidence interval.

**Figure 3:**
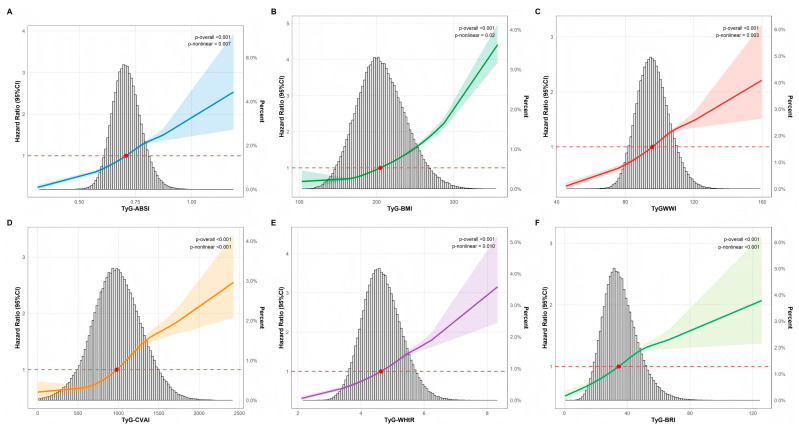
Dose–response relationships between six TyG-derived indices and incident cardiometabolic multimorbidity. Figure (**A**–**F**) show results for (**A**) TyG-ABSI, (**B**) TyG-BMI, (**C**) TyG-WWI, (**D**) TyG-CVAI, (**E**) TyG-WHtR, and (**F**) TyG-BRI. The solid lines represent the HR point estimates. The shaded areas indicate the 95% CI. The red dots mark the reference point for each index where the HR is 1.0. The horizontal dashed line represents the reference value of 1.0. The grey histograms in the background represent the population distribution, showing the percentage of participants as indicated by the right Y-axis. Note: Dose–response relationship of TyG-derived indices with incident cardiometabolic multimorbidity. Models were adjusted for age, gender, education status, marital status, smoking status, drinking status, PA, BMI, ALT, AST, SCr, TC, LDL-C, HDL-C, SBP, DBP, COPD, cancer, hyperlipidemia, and use of antihypertensive or glucose-lowering medication; variables constituting the respective TyG-derived index were excluded. Abbreviations: CI, confidence interval; TyG, triglyceride-glucose index; BMI, body mass index; WWI, weight-adjusted waist index; WHtR, waist-to-height ratio; HDL-C, high-density lipoprotein cholesterol; SBP, systolic blood pressure; DBP, diastolic blood pressure; SCr, serum creatinine; COPD, chronic obstructive pulmonary disease.

Over a median monitoring period of 4.3 years, we identified 7816 new CMM cases. As shown in Figure 2A–F and Table S1, the risk of CMM increased progressively with higher levels of TyG-ABSI, TyG-BMI, TyG-WWI, TyG-CVAI, TyG-WHtR, and TyG-BRI. In the fully adjusted analysis (Model 3), we used the lowest quartile (Q1) as the reference. Participants in the highest quartile (Q4) exhibited significantly elevated risks. Specifically, the hazard ratios (HRs) were: 1.657 (1.546–1.775), 2.146 (1.997–2.306), 1.737 (1.614–1.870), 1.922 (1.734–2.130), 2.150 (1.998–2.314), and 1.527 (1.393–1.673), respectively. Regarding survival analysis, Kaplan–Meier curves demonstrated a stepwise increase in cumulative CMM incidence from Q1 to Q4. The differences among quartiles were statistically significant (Figure 2G–L). Furthermore, Figure 3 depicts the dose–response patterns. RCS analysis confirmed significant overall associations for all six indices (*p*-overall < 0.001). Non-linear relationships were observed for all metrics. The strongest evidence for non-linearity was found for TyG-CVAI and TyG-BRI (*p*-non-linear < 0.001), followed by TyG-WWI (*p* = 0.003), TyG-ABSI (*p* = 0.007), TyG-WHtR (*p* = 0.010), and TyG-BMI (*p* = 0.02).

### 3.3. Predictive Value of TyG-Derived Indices in Incident CMM

In the ROC-curve analysis presented in Figure S1, TyG-BMI demonstrated the highest predictive accuracy for incident CMM among the six TyG-derived indices, yielding the largest AUC (0.623). The AUCs of the remaining indices ranked from high to low as follows: TyG-WHtR = 0.616, TyG-CVAI = 0.614, TyG-BRI = 0.602, TyG-WWI = 0.588, and TyG-ABSI = 0.573.

### 3.4. Subgroup and Sensitivity Analyses

Figure S2 and Table S6 present the subgroup and interaction analyses, stratified by age, gender, smoking status, drinking status, education status and PA. TyG-BMI, TyG-CVAI, TyG-WHtR, TyG-BRI exhibited significantly larger effect sizes in participants aged 65–75 years than in those ≥75 years (*p*-for-interaction < 0.05). Among the smoking-status strata, TyG-ABSI, TyG-WWI and TyG-BRI all exhibited amplified associations among ever- and current smokers (*p*-for-interaction < 0.05). In terms of educational attainment, only TyG-BMI demonstrated a significant interaction effect (*p*-interaction = 0.036). Subsequent analyses revealed a U-shaped pattern: HRs were substantially elevated among participants with no/limited schooling as well as those with tertiary education, compared with the medium-education group. In addition, across PA categories, only TyG-CVAI demonstrated a significant interaction (*p*-interaction = 0.021); the association was substantially stronger in the low-frequency group than in the high-frequency group. Collectively, these subgroup analyses underscore that the TyG-related indices are differentially influenced by age, smoking status, education status, and PA profiles, whereas gender and drinking status conferred no discernible effect modification (all *p* > 0.05).

To confirm the reliability of our main findings, we performed a series of sensitivity tests. Initially, we restricted the analysis to participants not receiving treatment for diabetes, hypertension, or dyslipidemia at baseline. The outcomes remained unchanged (Table S2). We also removed individuals with a follow-up duration shorter than one year, yielding similar observations (Table S3). Furthermore, the significant association persisted even after excluding participants with any pre-existing cardiometabolic or chronic diseases at baseline. These findings are presented in Table S4. Lastly, excluding those aged over 85 years did not alter the main conclusions (Table S5).

### 3.5. Mediation Analysis

**Figure 4:**
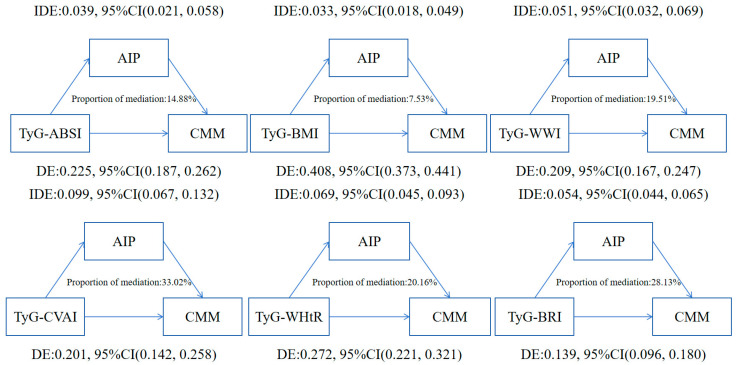
Mediation of the association between TyG-derived indices and the risk of cardiometabolic multimorbidity in the GOLD-Health cohort by AIP. Note: Models were adjusted for age, gender, education status, marital status, smoking status, drinking status, PA, BMI, ALT, AST, SCr, TC, LDL-C, HDL-C, SBP, DBP, COPD, cancer, hyperlipidemia, and use of antihypertensive or glucose-lowering medication; variables constituting the respective TyG-derived index were excluded. Abbreviations: DE, Direct effect; IDE, Indirect effect; AIP, atherogenic index of plasma; TyG-ABSI, triglyceride glucose, a body shape index; TyG-BMI, triglyceride glucose, body mass index; TyG-WWI, triglyceride, glucose, weight-adjusted waist index; TyG-WHtR, triglyceride, glucose, waist to height ratio; TyG-BRI, triglyceride glucose, Body Roundness Index; TyG-CVAI, triglyceride glucose, Chinese Visceral Adiposity.

Mediation analysis revealed that the AIP significantly conveys part of the effect of TyG-derived indices on incident CMM after full covariate adjustment. Among the six indices, TyG-CVAI exhibited the largest indirect effect, with 33.02% of its total association mediated by AIP, followed by TyG-BRI (28.13%) and TyG-WHtR (20.16%). Substantially smaller mediated proportions were observed for TyG-WWI (19.51%), TyG-ABSI (14.88%), and TyG-BMI (7.53%) (Figure 4 and Table S7).

## 4. Discussion

### 4.1. Primary Findings

This study is based on the large-scale prospective GOLD-Health cohort, comprehensively evaluating of six TyG-derived adiposity indices in relation to incident CMM among community-dwelling older adults. Our primary findings indicate that all six indices were independently and positively associated with the risk of developing CMM, with TyG-WHtR showing the strongest association; Although this indicator has limited efficacy when used alone to predict disease, it retains reference value in primary population risk screening given its significant correlation with disease risk. Furthermore, the AIP played a significant mediating role, explaining up to 33% of the association, suggesting it may represent a key pathway linking IR, dyslipidemia, and multimorbidity.

### 4.2. Comparison with Previous Research

Our findings are consistent with and extend previous evidence that combining the TyG index with anthropometric measures enhances cardiovascular risk prediction [32,33,34]. Despite prior studies focused on single cardiovascular disease outcomes or mortality in general populations [35,36,37,38], our study specifically focuses on CMM among community-dwelling older adults. Consistent with research from the CHARLS and NHANES cohorts [33,36], we found that composite indices like TyG-BMI and TyG-WHtR outperformed the traditional TyG index or adiposity measures alone. This may be because they can simultaneously reflect the combined status of insulin resistance and visceral fat accumulation, thereby enabling a more accurate assessment of risk. Our results showed a high hazard ratio but a modest AUC. The high hazard ratio shows that TyG-WHtR is a strong risk factor for CMM. The modest AUC indicates that the index is not a perfect predictive tool for individual patients. Nevertheless, these markers remain very practical for community screening. They offer a low-cost method to identify high-risk older adults in primary care. Combining these indices with established clinical risk scores might further enhance predictive accuracy. However, our primary goal was to evaluate their independent value as simple screening tools. Future studies can explore the added benefit of integrating these indices into existing clinical models.

### 4.3. Potential Biological Mechanisms

The innovation of this study lies in its conducting the first quantitative assessment of the mediating role of AIP. We found that AIP mediated a substantial proportion of the effect of TyG-derived indices on CMM. AIP, a logarithmically transformed ratio of molar concentrations of triglycerides to HDL-C [27,39], is a sensitive marker for the presence of small, dense LDL (sdLDL) particles [40,41]. We prioritized AIP for several biological reasons. The TyG index measures IR. High IR directly causes lipid disorders like high triglycerides and low HDL-C. AIP is a robust marker for this specific pattern [41]. It directly reflects the size of sdLDL particles [40]. These particles are the most direct link between IR and vascular damage. Other factors like blood pressure and inflammation also contribute to CMM. However, they represent different biological pathways. Our study focused on this metabolic-lipid axis to clarify the direct impact of IR. One technical consideration is the shared triglyceride component between TyG-derived indices and AIP. This mathematical overlap might lead to an overestimation of the mediation proportion. However, these two indices represent distinct biological stages. TyG serves as a surrogate for IR, while AIP specifically captures the resulting lipid remodeling and sdLDL size. Therefore, this analysis still offers important insights into the metabolic-lipid pathway leading to CMM. The mechanism linking these indices to CMM likely involves a cascade of lip toxicity and chronic inflammation. IR in adipose tissue promotes the release of free fatty acids and increases hepatic very low-density lipoprotein cholesterol (VLDLC) production [42], leading to the characteristic dyslipidemia phenotype (high TG, low HDL) [43]. This environment fosters the formation of atherogenic sdLDL particles, which are prone to oxidation and infiltration into the arterial wall, triggering endothelial dysfunction and atherosclerosis [44,45]. Furthermore, visceral adiposity, acts as an active endocrine organ, secreting pro-inflammatory cytokines (e.g., IL-6, TNF-α) that exacerbate systemic inflammation and thrombotic risk [46]. Our mediation analysis provides empirical support for this causal pathway, demonstrating that interventions targeting atherosclerotic dyslipidemia—such as early screening for high-risk populations, enhanced health education, and promotion of physical activity—can partially mitigate the risk of coronary heart disease associated with insulin-resistant obesity.

### 4.4. Strengths and Limitations

This study presents the following strengths. Firstly, the study’s prospective design, coupled with a large cohort of community-dwelling older adults from southern China, supports the robustness of the findings within this population. Secondly, combining the TyG index with obesity-related anthropometric measures yields potentially stronger associations and improved predictive performance for CMM, with TyG-WHtR showing a suggestive correlation and TyG-BMI exhibiting relatively better discriminative accuracy. Moreover, the availability of granular biomarker data enables a deeper dissection of the mechanistic pathways through which TyG-derived indices precipitate cardiometabolic multimorbidity, thereby furnishing invaluable insights for future experimental research into the underlying biology. In addition, comprehensive subgroup and sensitivity analyses corroborate the robustness and internal validity of our findings. Finally, to the best of our knowledge, this is the first study to use the AIP as a mediator to explore the mechanisms linking TyG-derived indices with the onset of CMM.

Nonetheless, this study has certain limitations. Firstly, our findings are based on older Chinese adults in southern China. This population has unique dietary patterns and a specific healthcare system. These factors may influence metabolic health differently than in other regions. Therefore, our results should be interpreted with caution when applied to diverse ethnic groups. Further research in other populations is needed to confirm these associations. Secondly, we used only baseline data to evaluate the indices as early predictors. This design focuses on the practical value of a single screening in primary care. We did not explore how changes in IR or adiposity over time affect the risk. Although we collected some repeated measures in 2020 and 2021, the data were not complete for the entire population. To ensure a robust analysis with the largest possible sample size, we prioritized the high-quality baseline data. This helps identify high-risk individuals at the earliest stage. However, using only baseline data can lead to regression dilution bias. This happens when participants change their metabolic status during follow-up. For example, a high-risk person who improves their lifestyle may remain healthy, which lowers the calculated hazard ratio. This bias results in an underestimation of the true associations. Future studies should use more complete repeated data to track metabolic changes. Additionally, we lacked data on structured cardiovascular rehabilitation programs and inflammatory markers, which might influence the progression of CMM. Furthermore, this study focused solely on the AIP as the mediator; other potential mediating factors remain to be explored. Finally, while the HRs demonstrate a strong risk association, the modest AUC values suggest that TyG-derived indices should be viewed as complementary screening tools rather than primary predictive criteria. Their main clinical utility lies in initial risk stratification within community settings. They offer a practical way to identify high-priority individuals for more intensive follow-up, especially when more expensive predictive resources are limited. Despite these acknowledged limitations, our study furnishes valuable evidence delineating the associations between TyG-derived indices and the incidence of CMM.

### 4.5. Future Directions

Future research should focus on several key areas. First, we need to study gender and age differences more deeply. Men and women may show different metabolic risks as they age. Second, future studies should explore the interaction effects between these factors and TyG-derived indices. For example, the predictive power of these indices might change depending on a person’s gender or age group. Third, research should use longer follow-up periods to track long-term health outcomes. Finally, including younger populations and diverse ethnic groups will help test the generalizability of our findings.

### 4.6. Take-Home Messages

Clinicians should consider using TyG-derived indices in routine health checks for older adults. These indices, especially TyG-WHtR and TyG-BMI, are low-cost and easy to obtain. They help identify individuals at high risk for multiple cardiometabolic diseases early. Monitoring both IR and atherogenic dyslipidemia is essential for early prevention. Early intervention for these high-risk patients can reduce the long-term burden of chronic diseases.

## 5. Conclusions

In summary, TyG-derived indices, particularly TyG-WHtR and TyG-BMI, are robust predictors of incident CMM in older adults. These readily calculable metrics can serve as cost-effective tools for risk stratification in primary care. Furthermore, the significant mediating role of AIP highlights atherogenic dyslipidemia as a key therapeutic target for preventing the progression from metabolic risk factors to multimorbidity.

## Figures and Tables

**Table 1:** Baseline characteristics of the study individuals according to cardiometabolic multimorbidity.

Characteristic	Overall	Non-CMM	CMM	*p*-Value
*n* (%)	304,586	296,770	7816	
Age, year	71.98 ± 6.25	71.99 ± 6.27	71.53 ± 5.62	0.006
Height, m	1.57 ± 0.08	1.57 ± 0.08	1.57 ± 0.08	0.762
Weight, kg	58.01 ± 9.63	57.94 ± 9.62	60.70 ± 9.74	<0.001
FBG, mmol/L	5.39 ± 0.92	5.38 ± 0.91	5.82 ± 1.10	<0.001
ALT, U/L	20.31 ± 10.17	20.26 ± 10.11	22.46 ± 11.87	<0.001
AST, U/L	23.05 ± 7.39	23.04 ± 7.37	23.38 ± 8.14	0.204
SCr, μmol/L	81.75 ± 27.22	81.74 ± 27.26	82.18 ± 25.56	0.001
TC, mmol/L	5.45 ± 1.09	5.45 ± 1.09	5.46 ± 1.10	0.420
TG, mmol/L	1.63 ± 0.78	1.63 ± 0.78	1.81 ± 0.86	<0.001
LDL-C, mmol/L	3.32 ± 0.94	3.31 ± 0.94	3.36 ± 0.96	<0.001
HDL-C, mmol/L	1.42 ± 0.35	1.43 ± 0.35	1.37 ± 0.33	<0.001
DBP, mmol/L	78.10 ± 9.45	78.06 ± 9.45	79.36 ± 9.38	<0.001
SBP, mmol/L	135.50 ± 16.90	135.43 ± 16.91	138.19 ± 16.17	<0.001
WC, cm	83.43 ± 8.65	83.35 ± 8.64	86.19 ± 8.69	<0.001
BMI, kg/m^2^	23.59 ± 3.15	23.56 ± 3.15	24.68 ± 3.23	<0.001
BRI	4.08 ± 1.20	4.07 ± 1.20	4.43 ± 1.24	<0.001
ABSI	0.08 ± 0.01	0.08 ± 0.01	0.08 ± 0.01	<0.001
CVAI	111.74 ± 32.19	111.45 ± 32.16	122.79 ± 31.23	<0.001
WHtR	0.53 ± 0.06	0.53 ± 0.06	0.55 ± 0.06	<0.001
TyG	8.75 ± 0.47	8.74 ± 0.47	8.92 ± 0.49	<0.001
TyG-ABSI	0.71 ± 0.06	0.71 ± 0.06	0.73 ± 0.06	<0.001
TyG-WHtR	4.67 ± 0.62	4.67 ± 0.61	4.92 ± 0.63	<0.001
TyG-BMI	206.64 ± 31.85	206.28 ± 31.73	220.40 ± 33.34	<0.001
TyG-BRI	35.78 ± 11.07	35.68 ± 11.03	39.65 ± 11.62	<0.001
TyG-CVAI	983.13 ± 306.42	980.03 ± 305.83	1100.87 ± 305.81	<0.001
TyG-WWI	96.28 ± 9.55	96.20 ± 9.54	99.10 ± 9.52	<0.001
Gender, *n* (%)				0.033
Male	120,154 (39%)	117,162 (39%)	2992 (38%)	
Female	184,432 (61%)	179,608 (61%)	4824 (62%)	
Education Status, *n* (%)				<0.001
High school	50,886 (17%)	49,703 (17%)	1183 (15%)	
Illiterate	33,762 (11%)	32,885 (11%)	877 (11%)	
Middle school	57,691 (19%)	56,083 (19%)	1608 (21%)	
Primary school	96,122 (32%)	93,420 (31%)	2702 (35%)	
University or higher	66,125 (22%)	64,679 (22%)	1446 (19%)	
Married Status, *n* (%)				0.215
Other	37,028 (12%)	36,042 (12%)	986 (13%)	
Married	267,558 (88%)	260,728 (88%)	6830 (87%)	
Smoking Status, *n* (%)				0.062
Never	259,610 (85%)	252,925 (85%)	6685 (86%)	
Current	33,356 (11%)	32,550 (11%)	806 (10%)	
Former	11,620 (4%)	11,295 (4%)	325 (4%)	
Drinking Status, *n* (%)				0.010
Never	287,506 (94%)	280,076 (94%)	7430 (95%)	
Current	17,080 (6%)	16,694 (6%)	386 (5%)	
Physical Activity, *n* (%)				<0.001
High	126,642 (42%)	123,263 (42%)	3379 (43%)	
Low	84,156 (28%)	82,312 (28%)	1844 (24%)	
Moderate	93,788 (31%)	91,195 (31%)	2593 (33%)	
Hypertension, *n* (%)	158,986 (52%)	153,982 (52%)	5004 (64%)	<0.001
GMS, *n* (%)				<0.001
NGR	193,431 (64%)	190,185 (64%)	3246 (42%)	
Pre-DM	95,361 (31%)	91,742 (31%)	3619 (46%)	
DM	15,794 (5%)	14,843 (5%)	951 (12%)	
Hyperlipoidemia, *n* (%)	136,970 (45%)	132,977 (45%)	3993 (51%)	<0.001
Stroke, *n* (%)	844 (0%)	744 (0%)	70 (1%)	<0.001
CHD, *n* (%)	3700 (1%)	3390 (1%)	310 (4%)	<0.001
COPD, *n* (%)	1780 (1%)	1713 (1%)	67 (1%)	0.002
Cancer, *n* (%)	4221 (1%)	4124 (1%)	97 (1%)	0.289
Antihypertensive Agents, *n* (%)	98,256 (32%)	94,545 (32%)	3711 (47%)	<0.001
Antidiabetic Agents, *n* (%)	8904 (3%)	8249 (3%)	655 (8%)	<0.001
Antilipidemic Agents, *n* (%)	4187 (1%)	3984 (1%)	203 (3%)	<0.001

Note: Data were expressed as the mean ± standard deviation (SD) or median (IQR) or number (proportion). Abbreviations: FBG, Fasting Blood Glucose; ALT, alanine aminotransferase; AST, aspartate aminotransferase; SCr, Serum Creatinine; TC, Total Cholesterol; TG, Triglycerides; HDL-C, high-density lipoprotein, cholesterol; LDL-C, low-density lipoprotein, cholesterol; SBP, systolic blood pressure; DBP, diastolic blood pressure; WC, waist circumference; BMI, body mass index; BRI, Body Roundness Index; ABSI, A Body shape index; CVAI, Chinese Visceral Adiposity Index; WHtR, waist-to-height ratio; TyG, triglyceride, glucose; TyG-ABSI, triglyceride glucose, a body shape index; TyG-BMI, triglyceride glucose, body mass index; TyG-WWI, triglyceride, glucose, weight-adjusted waist index; TyG-WHtR, triglyceride, glucose, waist to height ratio; TyG-BRI, triglyceride glucose, Body Roundness Index; TyG-CVAI, triglyceride glucose, Chinese Visceral Adiposity Index; GMS, Glucose Metabolism Status; NGR, Normal Glucose Regulation; Pre-DM, Prediabetes Mellitus; DM, Diabetes Mellitus; CHD, coronary heart disease; COPD, Chronic Obstructive Pulmonary Disease.

## Data Availability

The data underlying this article cannot be shared publicly due to the privacy of individuals who participated in the study. The data will be shared on reasonable request to the corresponding author.

## Supplementary Materials

The following supporting information can be downloaded at: https://www.mdpi.com/article/10.3390/nu18060985/s1, Figure S1: Comparative predictive performance of six TyG-derived indices for incident cardiometabolic multimorbidity; Figure S2: Subgroup analyses of the associations between six TyG-derived indices and incident cardiometabolic multimorbidity; Table S1: Associations between TyG-derived indices and risk of cardiometabolic multimorbidity in the GOLD-Health cohort; Table S2: Sensitivity analysis excluding participants receiving antihyperglycemic, antilipidemic, or antihypertensive treatment; Table S3: Sensitivity analysis excluding participants with follow-up duration less than 1 year; Table S4: Sensitivity analysis excluding participants with pre-existing cardiometabolic or chronic diseases; Table S5: Sensitivity analysis excluding participants aged over 85 years at baseline; Table S6: Subgroup analyses of the associations between TyG-derived indices and incident cardiometabolic multimorbidity; Table S7: Mediation of the associations between TyG-derived indices and the risk of cardiometabolic multimorbidity in the GOLD-Health cohort by AIP.

## Abbreviations

The following abbreviations are used in this manuscript:

CMD	cardiometabolic disease
CMM	cardiometabolic multimorbidity
FBG	Fasting Blood Glucose
ALT	alanine aminotransferase
AST	aspartate aminotransferase
SCr	Serum Creatinine
TyG	triglyceride, glucose
BRI	Body Roundness Index
ABSI	A body shape index
CVAI	Chinese Visceral Adiposity Index
WHtR	waist to height ratio
GMS	Glucose Metabolism Status
NGR	Normal Glucose Regulation
Pre-DM	Prediabetes Mellitus
DM	Diabetes Mellitus
COPD	Chronic Obstructive Pulmonary Disease
CHD	Coronary Heart Disease
