# Supplementary material for: Associations of TyG-Derived Indices with Cardiometabolic Multimorbidity Risk in Community-Dwelling Older Adults: A Longitudinal Analysis Based on the GOLD-Health Cohort
Source: Nutrients. 2026 Mar 19;18(6):985. doi: 10.3390/nu18060985 (PMC13029052; doi:10.3390/nu18060985)
Supplement: Supplementary file 1 [file nutrients-18-00985-s001.zip › nutrients-4178921-supplementary.pdf]

Supplementary material

Figure S1 Comparative predictive performance of six TyG-derived indices for incident cardiometabolic multimorbidity

Figure S2. Subgroup analyses of the associations between six TyG-derived indices and incident cardiometabolic multimorbidity

Table S1. Associations between TyG-derived indices and risk of cardiometabolic multimorbidity in the GOLD-Health cohort

Table S2. Sensitivity analysis excluding participants receiving antihyperglycemic, antilipidemic, or antihypertensive treatment

Table S3. Sensitivity analysis excluding participants with follow-up duration less than 1 year

Table S4. Sensitivity analysis excluding participants with pre-existing cardiometabolic or chronic diseases

Table S5. Sensitivity analysis excluding participants aged over 85 years at baseline

Table S6. Subgroup analyses of the associations between TyG-derived indices and incident cardiometabolic multimorbidity

Table S7. Mediation of the associations between TyG-derived indices and the risk of cardiometabolic multimorbidity in the GOLD-Health cohort by AIP

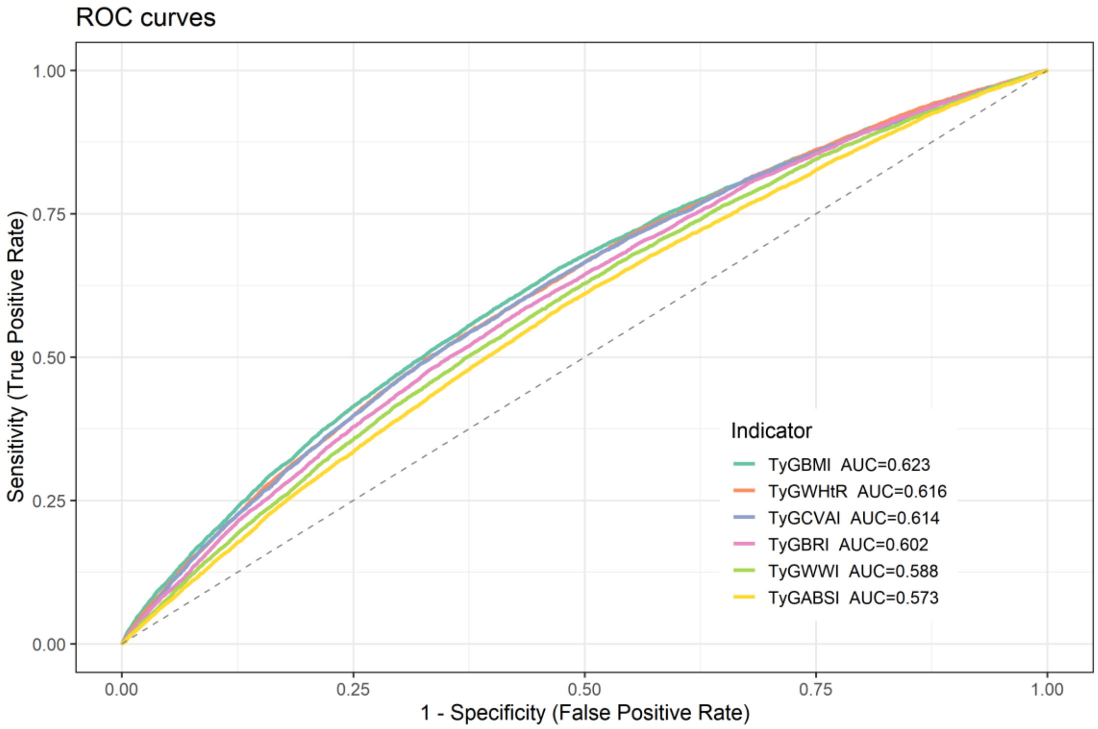

Figure S1 Comparative predictive performance of six TyG-derived indices for incident cardiometabolic multimorbidity.

Note: Abbreviations: TyG- ABSI, triglyceride glucose, a Body shape index; TyG-BMI, triglyceride glucose, body mass index; TyG-WWI, triglyceride, glucose, weight-adjusted waist index; TyG- WHtR, triglyceride, glucose, waist to height ratio; TyG- BRI, triglyceride glucose, Body Roundness Index; TyG-CVAI, triglyceride glucose, Chinese Visceral Adiposity Index.

TyG-ABSI

Subgroup	Events	Adjusted HR (95% CI)	P-value	P for interaction
Overall	7816	1.401 (1.336-1.470)	<0.001	
Gender				0.201
Female	4824	1.354 (1.274-1.440)	<0.001	
Male	2992	1.470 (1.362-1.587)	<0.001	
Age				0.085
65-75	5855	1.432 (1.355-1.513)	<0.001	
75+	1961	1.305 (1.185-1.436)	<0.001	
Smoking status				0.006
Never	6685	1.359 (1.291-1.431)	<0.001	
Current	806	1.660 (1.426-1.934)	<0.001	
Former	325	1.763 (1.389-2.238)	<0.001	
Drinking status				0.299
Never	7430	1.397 (1.330-1.467)	<0.001	
Current	386	1.507 (1.213-1.873)	<0.001	
Education status				0.216
Middle school	1608	1.444 (1.300-1.604)	<0.001	
University or higher	1446	1.515 (1.355-1.695)	<0.001	
Illiterate	877	1.498 (1.294-1.735)	<0.001	
High school	1183	1.298 (1.151-1.464)	<0.001	
Primary school	2702	1.331 (1.227-1.444)	<0.001	
PA				0.462
high	3379	1.441 (1.340-1.551)	<0.001	
moderate	2593	1.344 (1.236-1.460)	<0.001	
low	1844	1.395 (1.266-1.538)	<0.001	

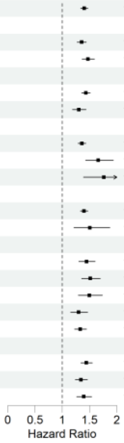

TyG-BMI

Subgroup	Events	Adjusted HR (95% CI)	P-value	P for interaction
Overall	7816	1.646 (1.565-1.731)	<0.001	
Gender				0.658
Female	4824	1.665 (1.561-1.776)	<0.001	
Male	2992	1.613 (1.489-1.749)	<0.001	
Age				<0.001
65-75	5855	1.722 (1.623-1.828)	<0.001	
75+	1961	1.461 (1.329-1.607)	<0.001	
Smoking status				0.425
Never	6685	1.648 (1.561-1.741)	<0.001	
Current	806	1.664 (1.424-1.944)	<0.001	
Former	325	1.486 (1.157-1.909)	0.002	
Drinking status				0.963
Never	7430	1.652 (1.569-1.739)	<0.001	
Current	386	1.545 (1.228-1.944)	<0.001	
Education status				0.036
Middle school	1608	1.546 (1.386-1.726)	<0.001	
University or higher	1446	1.834 (1.626-2.069)	<0.001	
Illiterate	877	1.822 (1.568-2.119)	<0.001	
High school	1183	1.520 (1.338-1.726)	<0.001	
Primary school	2702	1.601 (1.469-1.745)	<0.001	
PA				0.004
high	3379	1.533 (1.420-1.655)	<0.001	
moderate	2593	1.678 (1.537-1.832)	<0.001	
low	1844	1.801 (1.625-1.997)	<0.001	

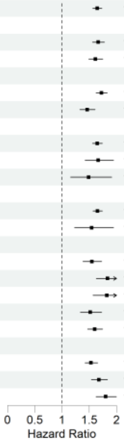

TyG-WWI

Subgroup	Events	Adjusted HR (95% CI)	P-value	P for interaction
Overall	7816	1.366 (1.299-1.437)	<0.001	
Gender				0.478
Female	4824	1.334 (1.249-1.425)	<0.001	
Male	2992	1.415 (1.309-1.530)	<0.001	
Age				0.126
65-75	5855	1.374 (1.297-1.455)	<0.001	
75+	1961	1.329 (1.201-1.471)	<0.001	
Smoking status				0.014
Never	6685	1.329 (1.259-1.403)	<0.001	
Current	806	1.653 (1.418-1.928)	<0.001	
Former	325	1.505 (1.186-1.912)	<0.001	
Drinking status				0.366
Never	7430	1.365 (1.296-1.437)	<0.001	
Current	386	1.413 (1.134-1.760)	0.002	
Education status				0.144
Middle school	1608	1.369 (1.228-1.527)	<0.001	
University or higher	1446	1.478 (1.314-1.663)	<0.001	
Illiterate	877	1.417 (1.209-1.659)	<0.001	
High school	1183	1.253 (1.106-1.418)	<0.001	
Primary school	2702	1.348 (1.236-1.470)	<0.001	
PA				0.274
high	3379	1.414 (1.310-1.527)	<0.001	
moderate	2593	1.290 (1.182-1.408)	<0.001	
low	1844	1.380 (1.244-1.529)	<0.001	

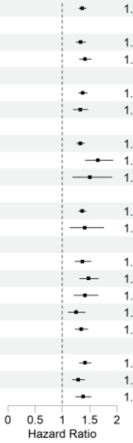

TyG-CVAI

Subgroup	Events	Adjusted HR (95% CI)	P-value	P for interaction
Overall	7816	1.379 (1.295-1.467)	<0.001	
Gender				0.270
Female	4824	1.438 (1.320-1.566)	<0.001	
Male	2992	1.319 (1.202-1.447)	<0.001	
Age				<0.001
65-75	5855	1.412 (1.314-1.516)	<0.001	
75+	1961	1.217 (1.073-1.380)	0.002	
Smoking status				0.364
Never	6685	1.388 (1.296-1.486)	<0.001	
Current	806	1.388 (1.155-1.668)	<0.001	
Former	325	1.240 (0.932-1.649)	0.140	
Drinking status				0.328
Never	7430	1.384 (1.298-1.476)	<0.001	
Current	386	1.349 (1.034-1.759)	0.027	
Education status				0.417
Middle school	1608	1.373 (1.199-1.572)	<0.001	
University or higher	1446	1.412 (1.220-1.633)	<0.001	
Illiterate	877	1.375 (1.129-1.675)	0.002	
High school	1183	1.392 (1.193-1.625)	<0.001	
Primary school	2702	1.368 (1.228-1.524)	<0.001	
PA				0.021
high	3379	1.382 (1.257-1.520)	<0.001	
moderate	2593	1.293 (1.161-1.440)	<0.001	
low	1844	1.499 (1.318-1.707)	<0.001	

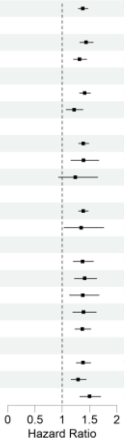

TyG-WHtR

Subgroup	Events	Adjusted HR (95% CI)	P-value	P for interaction
Overall	7816	1.356 (1.280-1.437)	<0.001	
Gender				0.734
Female	4824	1.332 (1.235-1.437)	<0.001	
Male	2992	1.396 (1.275-1.528)	<0.001	
Age				0.006
65-75	5855	1.366 (1.277-1.461)	<0.001	
75+	1961	1.319 (1.177-1.477)	<0.001	
Smoking status				0.096
Never	6685	1.323 (1.243-1.408)	<0.001	
Current	806	1.489 (1.245-1.782)	<0.001	
Former	325	1.869 (1.408-2.481)	<0.001	
Drinking status				0.315
Never	7430	1.354 (1.276-1.436)	<0.001	
Current	386	1.426 (1.101-1.847)	0.007	
Education status				0.106
Middle school	1608	1.396 (1.231-1.585)	<0.001	
University or higher	1446	1.455 (1.270-1.667)	<0.001	
Illiterate	877	1.419 (1.186-1.698)	<0.001	
High school	1183	1.309 (1.135-1.510)	<0.001	
Primary school	2702	1.291 (1.168-1.427)	<0.001	
PA				0.355
high	3379	1.381 (1.265-1.508)	<0.001	
moderate	2593	1.331 (1.204-1.472)	<0.001	
low	1844	1.338 (1.187-1.509)	<0.001	

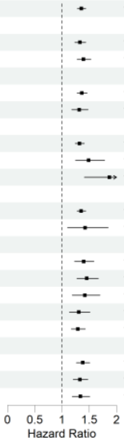

TyG-BRI

Subgroup	Events	Adjusted HR (95% CI)	P-value	P for interaction
Overall	7816	1.208 (1.141-1.280)	<0.001	
Gender				0.141
Female	4824	1.155 (1.071-1.245)	<0.001	
Male	2992	1.293 (1.179-1.417)	<0.001	
Age				0.002
65-75	5855	1.222 (1.143-1.307)	<0.001	
75+	1961	1.156 (1.032-1.295)	0.012	
Smoking status				0.001
Never	6685	1.153 (1.083-1.227)	<0.001	
Current	806	1.550 (1.293-1.858)	<0.001	
Former	325	1.811 (1.359-2.412)	<0.001	
Drinking status				0.594
Never	7430	1.211 (1.142-1.286)	<0.001	
Current	386	1.162 (0.896-1.506)	0.257	
Education status				0.084
Middle school	1608	1.231 (1.085-1.397)	0.001	
University or higher	1446	1.307 (1.142-1.497)	<0.001	
Illiterate	877	1.144 (0.958-1.367)	0.138	
High school	1183	1.118 (0.969-1.290)	0.127	
Primary school	2702	1.216 (1.100-1.344)	<0.001	
PA				0.057
high	3379	1.183 (1.083-1.291)	<0.001	
moderate	2593	1.198 (1.084-1.325)	<0.001	
low	1844	1.272 (1.128-1.435)	<0.001	

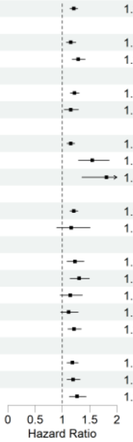

Figure S2. Subgroup analyses of the associations between six TyG-derived indices and incident cardiometabolic multimorbidity.

Note: Multivariable Cox models were adjusted for age, gender, education status, marital status, smoking status, drinking status, PA, BMI, ALT, AST, SCr, TC, LDL-C, HDL-C, SBP, DBP, COPD, cancer, hyperlipidemia, and use of antihypertensive or glucose-lowering medication (variables constituting the respective TyG-derived index were excluded).

Abbreviations: PA, physical activity; BMI, body mass index; ALT, alanine aminotransferase; AST, aspartate aminotransferase; SCr, serum creatinine; TC, total cholesterol; LDL-C, low-density lipoprotein cholesterol; HDL-C, high-density lipoprotein cholesterol; SBP, systolic blood pressure; DBP, diastolic blood pressure; COPD, chronic obstructive pulmonary disease; HR, hazard ratio; CI, confidence interv

1

Table S1. Associations between TyG-derived indices and risk of cardiometabolic multimorbidity in the GOLD-Health cohort

Groups	Event, n	Model 1		Model 2		Model 3	
		HR(95%CI)	*P*	HR(95%CI)	*P*	HR(95%CI)	*P*
TyG-ABSI Quartile							
Q1	1375	*Ref*		*Ref*		*Ref*	
Q2	1688	1.242 (1.161-1.330)	<0.001	1.246 (1.164-1.333)	<0.001	1.155 (1.079-1.236)	<0.001
Q3	2157	1.591 (1.490-1.698)	<0.001	1.606 (1.505-1.715)	<0.001	1.412 (1.321-1.509)	<0.001
Q4	2596	1.924 (1.803-2.052)	<0.001	1.975 (1.850-2.108)	<0.001	1.657 (1.546-1.775)	<0.001
P for trend			<0.001		<0.001		<0.001
TyG-BMI Quartile							
Q1	1107	*Ref*		*Ref*		*Ref*	
Q2	1440	1.366 (1.269-1.470)	<0.001	1.356 (1.260-1.460)	<0.001	1.201 (1.115-1.294)	<0.001
Q3	2077	1.961 (1.829-2.103)	<0.001	1.942 (1.811-2.082)	<0.001	1.568 (1.459-1.686)	<0.001
Q4	3192	3.003 (2.810-3.210)	<0.001	2.968 (2.776-3.173)	<0.001	2.146 (1.997-2.306)	<0.001
P for trend			<0.001		<0.001		<0.001
TyG-WWI Quartile							
Q1	1225	*Ref*		*Ref*		*Ref*	
Q2	1698	1.398 (1.304-1.499)	<0.001	1.427 (1.331-1.530)	<0.001	1.238 (1.154-1.328)	<0.001
Q3	2133	1.734 (1.622-1.855)	<0.001	1.809 (1.690-1.937)	<0.001	1.428 (1.331-1.532)	<0.001
Q4	2760	2.262 (2.119-2.416)	<0.001	2.453 (2.291-2.627)	<0.001	1.737 (1.614-1.870)	<0.001
P for trend			<0.001		<0.001		<0.001
TyG-CVAI Quartile							

Q1	1122	*Ref*		*Ref*		*Ref*	
Q2	1523	1.421 (1.322-1.528)	<0.001	1.485 (1.380-1.598)	<0.001	1.245 (1.152-1.345)	<0.001
Q3	2108	1.965 (1.834-2.106)	<0.001	2.113 (1.969-2.267)	<0.001	1.550 (1.424-1.687)	<0.001
Q4	3063	2.815 (2.633-3.008)	<0.001	3.113 (2.907-3.334)	<0.001	1.922 (1.734-2.130)	<0.001
P for trend			<0.001		<0.001		<0.001
TyG-WHtR Quartile							
Q1	1097	*Ref*		*Ref*		*Ref*	
Q2	1554	1.457 (1.356-1.567)	<0.001	1.473 (1.370-1.584)	<0.001	1.290 (1.199-1.389)	<0.001
Q3	2098	1.951 (1.821-2.092)	<0.001	1.998 (1.863-2.143)	<0.001	1.599 (1.488-1.719)	<0.001
Q4	3067	2.846 (2.662-3.042)	<0.001	2.981 (2.784-3.191)	<0.001	2.150 (1.998-2.314)	<0.001
P for trend			<0.001		<0.001		<0.001
TyG-BRI Quartile							
Q1	1151	*Ref*		*Ref*		*Ref*	
Q2	1659	1.461 (1.360-1.569)	<0.001	1.472 (1.370-1.582)	<0.001	1.191 (1.105-1.283)	<0.001
Q3	2090	1.870 (1.746-2.004)	<0.001	1.911 (1.782-2.048)	<0.001	1.325 (1.225-1.433)	<0.001
Q4	2916	2.559 (2.395-2.735)	<0.001	2.690 (2.513-2.880)	<0.001	1.527 (1.393-1.673)	<0.001
P for trend			<0.001		<0.001		<0.001

Note: Model 1 was unadjusted. Model 2 was adjusted for age and gender. Model 3 was adjusted for age, gender, education status, marital status, smoking status, drinking status, PA, BMI, ALT, AST, TC, LDL-C, HDL-C, SBP and DBP, SCr, COPD, cancer, hyperlipidemia, and use of antihypertensive or glucose-lowering medications; variables constituting the respective TyG-derived index were excluded. Kaplan–Meier curves show unadjusted cumulative incidence by quartiles.

Abbreviations: PA, physical activity; BMI, body mass index; ALT, alanine aminotransferase; AST, aspartate aminotransferase; TC, total cholesterol; LDL-C, low-density lipoprotein cholesterol; HDL-C, high-density lipoprotein cholesterol; SBP, systolic blood pressure; DBP, diastolic blood pressure; SCr, serum creatinine; COPD, chronic obstructive pulmonary disease.

Table S2. Sensitivity analysis excluding participants receiving antihyperglycemic, antilipidemic, or antihypertensive treatment

Groups	Model 1		Model 2		Model 3	
	HR (95%CI)	*P*	HR (95%CI)	*P*	HR (95%CI)	*P*
TyG-ABSI Quartile						
Q1	*Ref*		*Ref*		*Ref*	
Q2	1.174 (1.064-1.295)	0.001	1.174 (1.064-1.296)	0.001	1.112 (1.008-1.228)	0.035
Q3	1.441 (1.309-1.587)	<0.001	1.447 (1.314-1.593)	<0.001	1.325 (1.201-1.461)	<0.001
Q4	1.748 (1.589-1.924)	<0.001	1.769 (1.606-1.948)	<0.001	1.593 (1.439-1.764)	<0.001
TyG-BMI Quartile						
Q1	*Ref*		*Ref*		*Ref*	
Q2	1.338 (1.208-1.481)	<0.001	1.338 (1.209-1.482)	<0.001	1.229 (1.109-1.362)	<0.001
Q3	1.810 (1.640-1.999)	<0.001	1.812 (1.640-2.001)	<0.001	1.564 (1.411-1.733)	<0.001
Q4	2.791 (2.536-3.072)	<0.001	2.793 (2.536-3.077)	<0.001	2.224 (2.004-2.468)	<0.001
TyG-WWI Quartile						
Q1	*Ref*		*Ref*		*Ref*	
Q2	1.300 (1.177-1.435)	<0.001	1.320 (1.194-1.458)	<0.001	1.168 (1.056-1.291)	0.003
Q3	1.585 (1.438-1.748)	<0.001	1.634 (1.480-1.804)	<0.001	1.329 (1.200-1.471)	<0.001
Q4	2.079 (1.888-2.289)	<0.001	2.199 (1.989-2.431)	<0.001	1.638 (1.470-1.825)	<0.001
TyG-CVAI Quartile						
Q1	*Ref*		*Ref*		*Ref*	
Q2	1.392 (1.259-1.539)	<0.001	1.443 (1.303-1.598)	<0.001	1.223 (1.096-1.364)	<0.001
Q3	1.918 (1.739-2.114)	<0.001	2.027 (1.834-2.240)	<0.001	1.516 (1.340-1.715)	<0.001
Q4	2.631 (2.387-2.900)	<0.001	2.828 (2.558-3.127)	<0.001	1.798 (1.543-2.096)	<0.001

TyG-WHtR Quartile						
Q1	*Ref*		*Ref*		*Ref*	
Q2	1.425 (1.288-1.575)	<0.001	1.439 (1.301-1.592)	<0.001	1.311 (1.184-1.451)	<0.001
Q3	1.808 (1.637-1.996)	<0.001	1.844 (1.669-2.038)	<0.001	1.577 (1.422-1.748)	<0.001
Q4	2.619 (2.377-2.885)	<0.001	2.713 (2.457-2.996)	<0.001	2.161 (1.942-2.404)	<0.001
TyG-BRI Quartile						
Q1	*Ref*		*Ref*		*Ref*	
Q2	1.389 (1.257-1.534)	<0.001	1.400 (1.267-1.547)	<0.001	1.148 (1.035-1.275)	0.009
Q3	1.730 (1.568-1.909)	<0.001	1.761 (1.595-1.945)	<0.001	1.249 (1.115-1.399)	<0.001
Q4	2.337 (2.122-2.574)	<0.001	2.426 (2.196-2.679)	<0.001	1.415 (1.236-1.619)	<0.001

Note: Model 1 was unadjusted. Model 2 was adjusted for age and gender. Model 3 was adjusted for age, gender, education status, marital status, smoking status, drinking status, PA, BMI, ALT, AST, TC, LDL-C, HDL-C, SBP and DBP, SCr, COPD, cancer, hyperlipidemia, and use of antihypertensive or glucose-lowering medications; variables constituting the respective TyG-derived index were excluded.

Abbreviations: PA, physical activity; BMI, body mass index; ALT, alanine aminotransferase; AST, aspartate aminotransferase; TC, total cholesterol; LDL-C, low-density lipoprotein cholesterol; HDL-C, high-density lipoprotein cholesterol; SBP, systolic blood pressure; DBP, diastolic blood pressure; SCr, serum creatinine; COPD, chronic obstructive pulmonary disease.

Table S3. Sensitivity analysis excluding participants with follow-up duration less than 1 year

Groups	Model 1		Model 2		Model 3	
	HR(95%CI)	*P*	HR(95%CI)	*P*	HR(95%CI)	*P*
TyG-ABSI Quartile						
Q1	*Ref*		*Ref*		*Ref*	
Q2	1.240 (1.157-1.330)	<0.001	1.244 (1.160-1.333)	<0.001	1.151 (1.073-1.234)	<0.001
Q3	1.582 (1.480-1.692)	<0.001	1.598 (1.494-1.709)	<0.001	1.400 (1.307-1.499)	<0.001
Q4	1.926 (1.803-2.058)	<0.001	1.977 (1.850-2.114)	<0.001	1.650 (1.538-1.771)	<0.001
TyG-BMI Quartile						
Q1	*Ref*		*Ref*		*Ref*	
Q2	1.393 (1.292-1.503)	<0.001	1.384 (1.283-1.493)	<0.001	1.223 (1.133-1.320)	<0.001
Q3	1.966 (1.830-2.112)	<0.001	1.947 (1.811-2.092)	<0.001	1.566 (1.454-1.687)	<0.001
Q4	3.049 (2.848-3.265)	<0.001	3.015 (2.814-3.229)	<0.001	2.168 (2.013-2.335)	<0.001
TyG-WWI Quartile						
Q1	*Ref*		*Ref*		*Ref*	
Q2	1.398 (1.302-1.501)	<0.001	1.427 (1.329-1.533)	<0.001	1.227 (1.142-1.319)	<0.001
Q3	1.740 (1.624-1.865)	<0.001	1.816 (1.693-1.947)	<0.001	1.406 (1.308-1.511)	<0.001
Q4	2.261 (2.114-2.419)	<0.001	2.452 (2.286-2.630)	<0.001	1.683 (1.560-1.816)	<0.001
TyG-CVAI Quartile						
Q1	*Ref*		*Ref*		*Ref*	
Q2	1.419 (1.317-1.529)	<0.001	1.483 (1.376-1.599)	<0.001	1.240 (1.145-1.343)	<0.001
Q3	1.978 (1.843-2.123)	<0.001	2.127 (1.979-2.287)	<0.001	1.554 (1.425-1.695)	<0.001
Q4	2.823 (2.636-3.023)	<0.001	3.123 (2.911-3.351)	<0.001	1.915 (1.724-2.128)	<0.001

TyG-WHtR Quartile						
Q1	*Ref*		*Ref*		*Ref*	
Q2	1.454 (1.350-1.566)	<0.001	1.469 (1.364-1.583)	<0.001	1.284 (1.191-1.384)	<0.001
Q3	1.971 (1.836-2.116)	<0.001	2.018 (1.879-2.168)	<0.001	1.607 (1.493-1.730)	<0.001
Q4	2.844 (2.656-3.046)	<0.001	2.980 (2.778-3.196)	<0.001	2.132 (1.977-2.299)	<0.001
TyG-BRI Quartile						
Q1	*Ref*		*Ref*		*Ref*	
Q2	1.389 (1.257-1.534)	<0.001	1.400 (1.267-1.547)	<0.001	1.148 (1.035-1.275)	0.009
Q3	1.730 (1.568-1.909)	<0.001	1.761 (1.595-1.945)	<0.001	1.249 (1.115-1.399)	<0.001
Q4	2.337 (2.122-2.574)	<0.001	2.426 (2.196-2.679)	<0.001	1.415 (1.236-1.619)	<0.001

Note: Model 1 was unadjusted. Model 2 was adjusted for age and gender. Model 3 was adjusted for age, gender, education status, marital status, smoking status, drinking status, PA, BMI, ALT, AST, TC, LDL-C, HDL-C, SBP and DBP, SCr, COPD, cancer, hyperlipidemia, and use of antihypertensive or glucose-lowering medications; variables constituting the respective TyG-derived index were excluded.

Abbreviations: PA, physical activity; BMI, body mass index; ALT, alanine aminotransferase; AST, aspartate aminotransferase; TC, total cholesterol; LDL-C, low-density lipoprotein cholesterol; HDL-C, high-density lipoprotein cholesterol; SBP, systolic blood pressure; DBP, diastolic blood pressure; SCr, serum creatinine; COPD, chronic obstructive pulmonary disease.

Table S4. Sensitivity analysis excluding participants with pre-existing cardiometabolic or chronic diseases.

Groups	Model 1		Model 2		Model 3	
	HR(95%CI)	*P*	HR(95%CI)	*P*	HR(95%CI)	*P*
TyG-ABSI Quartile						
Q1	*Ref*		*Ref*		*Ref*	
Q2	1.448 (1.172-1.790)	<0.001	1.450 (1.173-1.792)	<0.001	1.428 (1.155-1.765)	0.001
Q3	1.317 (1.061-1.635)	0.013	1.324 (1.066-1.644)	0.011	1.295 (1.042-1.609)	0.020
Q4	1.671 (1.360-2.054)	<0.001	1.710 (1.390-2.104)	<0.001	1.737 (1.409-2.142)	<0.001
TyG-BMI Quartile						
Q1	*Ref*		*Ref*		*Ref*	
Q2	1.474 (1.165-1.864)	0.001	1.468 (1.160-1.857)	0.001	1.369 (1.080-1.734)	0.009
Q3	1.684 (1.339-2.117)	<0.001	1.673 (1.330-2.105)	<0.001	1.502 (1.189-1.897)	<0.001
Q4	2.579 (2.083-3.194)	<0.001	2.568 (2.070-3.184)	<0.001	2.220 (1.776-2.774)	<0.001
TyG-WWI Quartile						
Q1	*Ref*		*Ref*		*Ref*	
Q2	1.173 (0.942-1.461)	0.154	1.193 (0.957-1.486)	0.116	1.112 (0.892-1.387)	0.346
Q3	1.347 (1.089-1.667)	0.006	1.393 (1.124-1.728)	0.002	1.222 (0.983-1.519)	0.071
Q4	1.792 (1.466-2.192)	<0.001	1.938 (1.571-2.390)	<0.001	1.621 (1.307-2.011)	<0.001
TyG-CVAI Quartile						
Q1	*Ref*		*Ref*		*Ref*	
Q2	1.277 (1.120-1.455)	<0.001	1.320 (1.156-1.508)	<0.001	1.159 (1.003-1.338)	0.045
Q3	1.846 (1.625-2.098)	<0.001	1.941 (1.702-2.214)	<0.001	1.558 (1.321-1.838)	<0.001
Q4	2.553 (2.236-2.915)	<0.001	2.710 (2.364-3.107)	<0.001	1.943 (1.575-2.397)	<0.001

TyG-WHtR Quartile						
Q1	*Ref*		*Ref*		*Ref*	
Q2	1.321 (1.051-1.661)	0.017	1.337 (1.063-1.681)	0.013	1.267 (1.007-1.594)	0.044
Q3	1.449 (1.158-1.814)	0.001	1.485 (1.185-1.861)	<0.001	1.359 (1.082-1.707)	0.008
Q4	2.329 (1.895-2.864)	<0.001	2.455 (1.987-3.033)	<0.001	2.193 (1.765-2.726)	<0.001
TyG-BRI Quartile						
Q1	*Ref*		*Ref*		*Ref*	
Q2	1.245 (0.994-1.559)	0.057	1.256 (1.002-1.573)	0.048	1.071 (0.850-1.350)	0.561
Q3	1.408 (1.131-1.753)	0.002	1.439 (1.155-1.794)	0.001	1.112 (0.876-1.413)	0.383
Q4	2.086 (1.701-2.558)	<0.001	2.191 (1.778-2.700)	<0.001	1.432 (1.093-1.878)	0.009

Note: Model 1 was unadjusted. Model 2 was adjusted for age and gender. Model 3 was adjusted for age, gender, education status, marital status, smoking status, drinking status, PA, BMI, ALT, AST, TC, LDL-C, HDL-C, SBP and DBP, SCr, COPD, cancer, hyperlipidemia, and use of antihypertensive or glucose-lowering medications; variables constituting the respective TyG-derived index were excluded.

Abbreviations: PA, physical activity; BMI, body mass index; ALT, alanine aminotransferase; AST, aspartate aminotransferase; TC, total cholesterol; LDL-C, low-density lipoprotein cholesterol; HDL-C, high-density lipoprotein cholesterol; SBP, systolic blood pressure; DBP, diastolic blood pressure; SCr, serum creatinine; COPD, chronic obstructive pulmonary disease.

Table S5. Sensitivity analysis excluding participants aged over 85 years at baseline

Groups	Model 1		Model 2		Model 3	
	HR(95%CI)	*P*	HR(95%CI)	*P*	HR(95%CI)	*P*
TyG-ABSI Quartile						
Q1	*Ref*		*Ref*		*Ref*	
Q2	1.247 (1.163-1.336)	<0.001	1.250 (1.167-1.340)	<0.001	1.158 (1.080-1.241)	<0.001
Q3	1.609 (1.506-1.720)	<0.001	1.621 (1.516-1.732)	<0.001	1.422 (1.329-1.522)	<0.001
Q4	1.985 (1.858-2.120)	<0.001	2.017 (1.888-2.155)	<0.001	1.687 (1.573-1.811)	<0.001
TyG-BMI Quartile						
Q1	*Ref*		*Ref*		*Ref*	
Q2	1.364 (1.264-1.472)	<0.001	1.362 (1.262-1.470)	<0.001	1.203 (1.114-1.299)	<0.001
Q3	1.971 (1.835-2.118)	<0.001	1.969 (1.833-2.115)	<0.001	1.585 (1.471-1.706)	<0.001
Q4	3.015 (2.815-3.228)	<0.001	3.013 (2.813-3.227)	<0.001	2.169 (2.014-2.335)	<0.001
TyG-WWI Quartile						
Q1	*Ref*		*Ref*		*Ref*	
Q2	1.403 (1.307-1.506)	<0.001	1.434 (1.336-1.539)	<0.001	1.232 (1.146-1.323)	<0.001
Q3	1.758 (1.642-1.883)	<0.001	1.833 (1.710-1.965)	<0.001	1.419 (1.321-1.525)	<0.001
Q4	2.327 (2.176-2.488)	<0.001	2.500 (2.332-2.681)	<0.001	1.716 (1.592-1.851)	<0.001
TyG-CVAI Quartile						
Q1	*Ref*		*Ref*		*Ref*	
Q2	1.432 (1.331-1.542)	<0.001	1.489 (1.382-1.604)	<0.001	1.246 (1.152-1.349)	<0.001
Q3	2.009 (1.873-2.155)	<0.001	2.130 (1.983-2.288)	<0.001	1.559 (1.431-1.700)	<0.001
Q4	2.921 (2.730-3.126)	<0.001	3.151 (2.939-3.378)	<0.001	1.942 (1.749-2.156)	<0.001

TyG-WHtR Quartile						
Q1	*Ref*		*Ref*		*Ref*	
Q2	1.489 (1.382-1.604)	<0.001	1.510 (1.401-1.626)	<0.001	1.319 (1.224-1.422)	<0.001
Q3	1.982 (1.846-2.129)	<0.001	2.039 (1.898-2.191)	<0.001	1.626 (1.510-1.751)	<0.001
Q4	2.925 (2.731-3.132)	<0.001	3.071 (2.863-3.294)	<0.001	2.204 (2.044-2.376)	<0.001
TyG-BRI Quartile						
Q1	*Ref*		*Ref*		*Ref*	
Q2	1.490 (1.385-1.604)	<0.001	1.507 (1.401-1.622)	<0.001	1.215 (1.125-1.311)	<0.001
Q3	1.906 (1.776-2.045)	<0.001	1.954 (1.820-2.098)	<0.001	1.350 (1.245-1.462)	<0.001
Q4	2.627 (2.454-2.812)	<0.001	2.761 (2.575-2.961)	<0.001	1.559 (1.419-1.713)	<0.001

Note: Model 1 was unadjusted. Model 2 was adjusted for age and gender. Model 3 was adjusted for age, gender, education status, marital status, smoking status, drinking status, PA, BMI, ALT, AST, TC, LDL-C, HDL-C, SBP and DBP, SCr, COPD, cancer, hyperlipidemia, and use of antihypertensive or glucose-lowering medications; variables constituting the respective TyG-derived index were excluded.

Abbreviations: PA, physical activity; BMI, body mass index; ALT, alanine aminotransferase; AST, aspartate aminotransferase; TC, total cholesterol; LDL-C, low-density lipoprotein cholesterol; HDL-C, high-density lipoprotein cholesterol; SBP, systolic blood pressure; DBP, diastolic blood pressure; SCr, serum creatinine; COPD, chronic obstructive pulmonary disease.

Table S6. Subgroup analyses of the associations between TyG-derived indices and incident cardiometabolic multimorbidity

Subgroup	Event, n	Adjusted HR (95% CI) & P for interaction					
		TyG-ABSI	*P* for interaction	TyG-BMI	*P* for interaction	TyG-WWI	*P* for interaction
**Overall**	7816	1.401 (1.336-1.470)		1.646 (1.565-1.731)		1.366 (1.299-1.437)	
Gender			0.201		0.658		0.478
Female	4824	1.354 (1.274-1.440)		1.665 (1.561-1.776)		1.334 (1.249-1.425)	
Male	2992	1.470 (1.362-1.587)		1.613 (1.489-1.749)		1.415 (1.309-1.530)	
Age			0.085		<0.001		0.126
65-75	5855	1.432 (1.355-1.513)		1.722 (1.623-1.828)		1.374 (1.297-1.455)	
75+	1961	1.305 (1.185-1.436)		1.461 (1.329-1.607)		1.329 (1.201-1.471)	
Smoking status			0.006		0.425		0.014
Never	6685	1.359 (1.291-1.431)		1.648 (1.561-1.741)		1.329 (1.259-1.403)	
Current	806	1.660 (1.426-1.934)		1.664 (1.424-1.944)		1.653 (1.418-1.928)	
Former	325	1.763 (1.389-2.238)		1.486 (1.157-1.909)		1.505 (1.186-1.912)	
Drinking status			0.299		0.963		0.366
Never	7430	1.397 (1.330-1.467)		1.652 (1.569-1.739)		1.365 (1.296-1.437)	
Current	386	1.507 (1.213-1.873)		1.545 (1.228-1.944)		1.413 (1.134-1.760)	
Education status			0.216		0.036		0.144
Middle school	1608	1.444 (1.300-1.604)		1.546 (1.386-1.726)		1.369 (1.228-1.527)	
University or higher	1446	1.515 (1.355-1.695)		1.834 (1.626-2.069)		1.478 (1.314-1.663)	
Illiterate	877	1.498 (1.294-1.735)		1.822 (1.568-2.119)		1.417 (1.209-1.659)	
High school	1183	1.298 (1.151-1.464)		1.520 (1.338-1.726)		1.253 (1.106-1.418)	

Primary school	2702	1.331 (1.227-1.444)		1.601 (1.469-1.745)		1.348 (1.236-1.470)	
PA			0.462		0.004		0.274
High	3379	1.441 (1.340-1.551)		1.533 (1.420-1.655)		1.414 (1.310-1.527)	
Moderate	2593	1.344 (1.236-1.460)		1.678 (1.537-1.832)		1.290 (1.182-1.408)	
Low	1844	1.395 (1.266-1.538)		1.801 (1.625-1.997)		1.380 (1.244-1.529)	

**Subgroup**	**Event, n**	**Adjusted HR (95% *CI*) & P for interaction**					
		**TyG-CVAI**	***P* for interaction**	**TyG-WHtR**	***P* for interaction**	**TyG-BRI**	***P* for interaction**
**Overall**	7816	1.379 (1.295-1.467)		1.356 (1.280-1.437)		1.208 (1.141-1.280)	
Gender			0.270		0.734		0.141
Female	4824	1.438 (1.320-1.566)		1.332 (1.235-1.437)		1.155 (1.071-1.245)	
Male	2992	1.319 (1.202-1.447)		1.396 (1.275-1.528)		1.293 (1.179-1.417)	
Age			<0.001		0.006		0.002
65-75	5855	1.412 (1.314-1.516)		1.366 (1.277-1.461)		1.222 (1.143-1.307)	
75+	1961	1.217 (1.073-1.380)		1.319 (1.177-1.477)		1.156 (1.032-1.295)	
Smoking status			0.364		0.096		0.001
Never	6685	1.388 (1.296-1.486)		1.323 (1.243-1.408)		1.153 (1.083-1.227)	
Current	806	1.388 (1.155-1.668)		1.489 (1.245-1.782)		1.550 (1.293-1.858)	
Former	325	1.240 (0.932-1.649)		1.869 (1.408-2.481)		1.811 (1.359-2.412)	
Drinking status			0.328		0.315		0.594
Never	7430	1.384 (1.298-1.476)		1.354 (1.276-1.436)		1.211 (1.142-1.286)	
Current	386	1.349 (1.034-1.759)		1.426 (1.101-1.847)		1.162 (0.896-1.506)	

Education status			0.417		0.106		0.084
Middle school	1608	1.373 (1.199-1.572)		1.396 (1.231-1.585)		1.231 (1.085-1.397)	
University or higher	1446	1.412 (1.220-1.633)		1.455 (1.270-1.667)		1.307 (1.142-1.497)	
Illiterate	877	1.375 (1.129-1.675)		1.419 (1.186-1.698)		1.144 (0.958-1.367)	
High school	1183	1.392 (1.193-1.625)		1.309 (1.135-1.510)		1.118 (0.969-1.290)	
Primary school	2702	1.368 (1.228-1.524)		1.291 (1.168-1.427)		1.216 (1.100-1.344)	
PA			0.021		0.355		0.057
High	3379	1.382 (1.257-1.520)		1.381 (1.265-1.508)		1.183 (1.083-1.291)	
Moderate	2593	1.293 (1.161-1.440)		1.331 (1.204-1.472)		1.198 (1.084-1.325)	
Low	1844	1.499 (1.318-1.707)		1.338 (1.187-1.509)		1.272 (1.128-1.435)	

Note: Multivariable Cox models were adjusted for age, gender, education status, marital status, smoking status, drinking status, PA, BMI, ALT, AST, SCr, TC, LDL-C, HDL-C, SBP, DBP, COPD, cancer, hyperlipidemia, and use of antihypertensive or glucose-lowering medication (variables constituting the respective TyG-derived index were excluded).

Abbreviations: PA, physical activity; BMI, body mass index; ALT, alanine aminotransferase; AST, aspartate aminotransferase; SCr, serum creatinine; TC, total cholesterol; LDL-C, low-density lipoprotein cholesterol; HDL-C, high-density lipoprotein cholesterol; SBP, systolic blood pressure; DBP, diastolic blood pressure; COPD, chronic obstructive pulmonary disease; HR, hazard ratio; CI, confidence interval.

Table S7 Mediation of the associations between TyG-derived indices and the risk of cardiometabolic multimorbidity in the GOLD-Health cohort by AIP

Independent variable	Total effect Coeffcient (95%CI)	*P* value	Indirect effect Coeffcient (95%CI)	*P* value	Direct effect Coeffcient (95%CI)	*P* value	Proportion mediated (%)
TyG-ABSI	0.265 (0.233, 0.296)	<0.001	0.039 (0.021, 0.058)	<0.001	0.225 (0.187, 0.262)	<0.001	14.88
TyG-BMI	0.441 (0.411, 0.472)	< 0.001	0.033 (0.018, 0.049)	<0.001	0.408 (0.373, 0.441)	< 0.001	7.53
TyG-WWI	0.259 (0.225, 0.292)	< 0.001	0.051 (0.032, 0.069)	< 0.001	0.209 (0.167, 0.247)	< 0.001	19.51
TyG-CVAI	0.301 (0.251, 0.345)	< 0.001	0.099 (0.067, 0.132)	< 0.001	0.201 (0.142, 0.258)	< 0.001	33.02
TyG-WHtR	0.341 (0.297, 0.384)	< 0.001	0.069 (0.045, 0.093)	< 0.001	0.272 (0.221, 0.321)	< 0.001	20.16
TyG-BRI	0.194 (0.151, 0.233)	< 0.001	0.054 (0.044, 0.065)	< 0.001	0.139 (0.096, 0.180)	< 0.001	28.13

Note: Models were adjusted for age, gender, education status, marital status, smoking status, drinking status, PA, BMI, ALT, AST, SCr, TC, LDL-C, HDL-C, SBP, DBP, COPD, cancer, hyperlipidemia, and use of antihypertensive or glucose-lowering medication; variables constituting the respective TyG-derived index were excluded.

Abbreviations: AIP, atherogenic index of plasma; TyG- ABSI, triglyceride glucose, a Body shape index; TyG-BMI, triglyceride glucose, body mass index; TyG-WWI, triglyceride, glucose, weight-adjusted waist index; TyG- WHtR, triglyceride, glucose, waist to height ratio; TyG- BRI, triglyceride glucose, Body Roundness Index; TyG-CVAI, triglyceride glucose, Chinese Visceral Adiposity Index.
